# Sanguinarine Induces H_2_O_2_-Dependent Apoptosis and Ferroptosis in Human Cervical Cancer

**DOI:** 10.3390/biomedicines10081795

**Published:** 2022-07-26

**Authors:** Ameer Alakkal, Faisal Thayyullathil, Siraj Pallichankandy, Karthikeyan Subburayan, Anees Rahman Cheratta, Sehamuddin Galadari

**Affiliations:** 1 Cell Death Signaling Laboratory, Division of Science (Biology), Experimental Research Building, New York University Abu Dhabi, Abu Dhabi P.O. Box 129188, United Arab Emirates; aa6272@nyu.edu (A.A.); ftt1@nyu.edu (F.T.); sp191@nyu.edu (S.P.); ks188@nyu.edu (K.S.); arc10@nyu.edu (A.R.C.); 2 Department of Biochemistry, College of Medicine and Health Sciences, UAE University, Al Ain P.O. Box 17666, United Arab Emirates

**Keywords:** apoptosis, ferroptosis, LPO, labile iron, ROS, sanguinarine

## Abstract

Sanguinarine (SNG) is a benzophenanthridine alkaloid isolated mainly from *Sanguinaria canadensis*, *Chelidonium majus*, and *Macleaya cordata*. SNG is considered an antineoplastic agent based on its cytotoxic activity against various tumors. However, the exact molecular mechanism through which SNG mediates this activity has not been elucidated. Here, we report that SNG induces death in human cervical cancer (HeLa) cells through activation of two interdependent cell death pathways—apoptosis and ferroptosis. SNG-induced apoptosis was characterized by caspase activation and PARP cleavage, while ferroptosis involved solute carrier family 7 member 11 (SLC7A11) down-regulation, glutathione (GSH) depletion, iron accumulation, and lipid peroxidation (LPO). Interestingly, incubation with caspase inhibitor z-VAD-fmk not only inhibited the features of apoptosis, but also negated markers of SNG-induced ferroptosis. Similarly, pretreatment with ferroptosis inhibitor ferrostatin-1 (Fer-1), apart from rescuing cells from SNG-induced ferroptosis, also curbed the features of SNG-induced apoptosis. Our study implies that, together, apoptosis and ferroptosis act as partners in the context of SNG mediated tumor suppression in HeLa cells. Importantly, SNG increased the generation of reactive oxygen species (ROS), and ROS inhibition blocks the induction of both apoptosis and ferroptosis. These findings highlight the value of continued investigation into the potential use of SNG as an antineoplastic agent.

## 1. Introduction

The tremendous chemical diversity found in millions of plants, animals, microorganisms, and marine organisms is an attractive source of novel therapeutic molecules. Drugs isolated from natural sources have been used for centuries to treat a variety of ailments, including cancer. Over 60% of the clinically approved anticancer drugs are derived from natural compounds. Therefore, there is intense interest in investigating nature-based molecules to develop the next generation of improved and effective cancer treatments. Sanguinarine (SNG) is a quaternary benzophenanthridine alkaloid isolated mainly from *Sanguinaria canadensis,* but also from *Chelidonium majus* and *Macleaya cordata* [1]. It exhibits a wide array of pharmacological activities, including antimicrobial, anti-hypertensive, anti-inflammatory, and anticancer properties [1]. Numerous in vivo and in vitro studies have demonstrated that SNG exerts a significant inhibitory effect on several different cancers, including cancers of the brain [2], prostate [3], lung [4], colon [5], bladder [6], breast [7], pancreas [8], and human leukemia [9]. However, the molecular signaling mechanism involved in SNG-induced cervical cancer suppression is not fully understood, hence impeding its further development as a potential anticancer agent.

Most anticancer agents suppress cancer growth via inducing various programmed cell death pathways such as apoptosis, autophagy, necroptosis, and ferroptosis. Apoptosis, also known as type-I programmed cell death, is the most predominant tumor suppressive pathway activated by various anticancer agents [10]. Apoptosis can be triggered through either extrinsic (death receptor mediated) and/or intrinsic (mitochondrial-dependent) pathways [10]. Both these apoptotic pathways are initiated and executed by a family of cysteine proteases known as caspases. On the other hand, ferroptosis is a caspase-independent, but reactive oxygen species (ROS)-dependent non-apoptotic cell death caused by the accumulation of iron and lipid peroxides [11]. ROS including superoxide anions (O_2_∙–), hydrogen peroxide (H_2_O_2_), and hydroxyl radicals (∙OH) play a vital role in the induction of various cell death pathways, including apoptosis and ferroptosis in response to chemo- or radio-therapy [12]. Ferroptosis can be elicited pharmacologically by inhibiting the system Xc^−^, a cysteine−glutamate antiporter containing solute carrier family 7 member 11 (SLC7A11) as the functional subunit, that facilitates sufficient cystine entry in the cells. As cysteine is necessary for synthesizing the glutathione (GSH), inhibition of system Xc^−^ could lead to a depletion of intracellular GSH and decreased activity of glutathione peroxidase 4 (GPX4), leading to increasing the ROS formation and lipid peroxidation (LPO), triggering ferroptosis [13]. Emerging evidence has suggested that ferroptosis is a new therapeutic strategy to overcome drug resistance in cancer cells [14].

In the present study, we investigated the anticancer effect of SNG in human cervical cancer cells in vitro. We have found that SNG efficiently activates both apoptosis and ferroptosis simultaneously. Furthermore, we investigated the molecular signaling mechanism involved in SNG-induced cytotoxicity. We observed that SNG-induced apoptosis and ferroptosis are regulated by ROS, specifically H_2_O_2_-mediated SLC7A11 down regulation and GSH depletion. Therefore, our findings might provide new strategies for the comprehensive treatment of cervical cancer.

## 2. Materials and Methods

### 2.1. Chemicals and Antibodies

MTT (3-[4,5-dimethylthiazol-2-yl]-2,5-diphenyl tetrazolium bromide), SNG, GSH assay kit, iron assay kit, N-acetyl cysteine (NAC), superoxide dismutase (SOD), Trolox (6-hydroxy-2,5,7,8-tetramethylchromane-2-carboxylic acid), Hoechst 33342, ferrostatin-1 (Fer-1), deferoxamine (DFO), necrosulfonamide (NSA), bafilomycinA1 (BafA1), 2′,7′-Dichlorodihydrofluorescein diacetate (DCFH-DA), dimethyl sulfoxide (DMSO), anti-rabbit IgG (#A6154), and anti-mouse IgG (#A0412) were purchased from Sigma Chemical Co. (St. Louis, MO, USA). Dulbecco’s Modified Essential Medium (DMEM), phosphate buffered-saline (PBS), trypsin-EDTA, sodium pyruvate (Sod-Py), and fetal bovine serum (FBS) were purchased from Gibco BRL (Grand Island, NY, USA). Anti-actin (#sc-47778) (1:1000), and donkey anti-goat IgG (#sc-2056) antibodies were purchased from Santa Cruz Biotechnology Inc. (Santa Cruz, CA, USA). Anti-SLC7A11 (#12691S) (1:1000), anti-TFR1/CD71 (#13113S) (1:1000), anti-DMT1/SLC11A2 (#15083S) (1:1000), and anti-PARP (#9542) (1:1000) antibodies were from Cell Signaling Technology (Beverly, MA, USA). Image-iT™ LPO kit (#C10445) and Phen Green SK (P14313) were purchased from Molecular Probes-Invitrogen Life Technologies (Waltham, MA, USA). Anti-GPX4 (#125066) (1:1000) was from Abcam (Cambridge, MA, USA) and N-benzoyloxycarbonyl-Val-Ala-Asp fluoromethylketone (z-VAD-fmk) was from Enzo Life Sciences (Lausen, Switzerland).

### 2.2. Cell Culture Conditions and SNG Treatment

Human cervical cancer (HeLa) cells were procured from ATCC (Rockville, MD, USA). The cells were cultured in DMEM supplemented with 10% *v*/*v* heat-inactivated FBS, 25 IU/mL penicillin, and 25 μg/mL streptomycin in an incubator containing a humidified atmosphere of 95% air and 5% CO_2_ at 37 °C. The cells were checked quarterly for mycoplasma contamination using the first generation MycoAlert™ mycoplasma detection kit (Lonza, #: LT07-118). SNG stock solution (10 mM in DMSO) was prepared in a dark-colored bottle, from which the desired dilutions were made. Cells were grown to about 80% confluence and then treated with SNG at different concentrations and for different periods of time.

### 2.3. Cell Viability Assay

Cells (10,000 cells in 0.1 mL/well) grown in 96-well microtiter plates were treated with the required concentration of SNG for the required time period. After treatment, 25 μL of MTT (5 mg/mL) was added to each well and incubated for 2 h at 37 °C. The formazan crystals that formed were dissolved in 200 μL of DMSO and absorbance was measured at 570 nm using an EnSpire™ multimode plate reader (PerkinElmer, Waltham, MA, USA). The cytotoxicity was expressed as the percentage over control.

### 2.4. Live/Dead Assay

The number of live and dead cells were assessed using a live/dead assay kit (Thermo Scientific, Pleasanton, CA, USA) according to the manufacturer’s protocol. In brief, SNG exposed cells were incubated with a 2 μM calcein and 4 μM ethidium homodimer-1 mixture in the dark. After 30 min of incubation at 37 °C, live and dead cell images were captured using an IX73 inverted fluorescent microscope (Olympus, Tokyo, Japan).

### 2.5. Pretreatment with Scavengers/Inhibitors/Inducers

To investigate the involvement of various signaling pathways in SNG-induced cell death, 1 h prior to SNG treatment, the cells were treated with different scavengers/inhibitors/inducers such as z-VAD-fmk (50 μM), Fer-1 (20 μM), DFO (400 μM), Baf A1 (250 nM), NSA (5 μM), Trolox (2.5 mM), GSH (5 mM), NAC (5 mM), Sod-Py (5 mM), catalase (2000 U/mL), and SOD (500 U/mL).

### 2.6. Protein Lysate Preparation and Western Blot Analysis

After SNG treatment, Western blot analysis was performed as described previously [15]. The cells were washed twice with PBS and were lysed in a RIPA lysis buffer (50 mM Tris HCl pH 7.4, 1% NP-40, 40 mM NaF, 10 mM NaCl, 10 mM Na3VO4, 1 mM phenyl methyl sufonyl fluoride, 10 mM dithiothreitol, and EDTA-free protease inhibitor tablet). The cell lysates were centrifuged and the total protein was determined using a Bio-Rad protein assay. The lysates were mixed with a 6x loading buffer and boiled at 100 °C for 3 min. Samples at 30–50 μg/lane were resolved using SDS-PAGE and the separated proteins were transferred on to a nitrocellulose membrane by a wet transfer method using Bio-Rad electro transfer apparatus. Following the transfer, the blots were blocked with 5% non-fat milk in Tris-buffer saline containing 0.1% Tween-20. The blots were then incubated with primary antibodies followed by a secondary antibody. The protein bands were visualized using a Super Signal West Pico Chemiluminescence reagent (Thermo Scientific, Pleasanton, CA, USA).

### 2.7. Intracellular and Mitochondrial ROS Measurement

Intracellular ROS generation was measured using an oxidation-sensitive fluorescent probe DCFH-DA as described previously [2]. Briefly, cells pre-incubated with 25 μM DCFH-DA (30 min in dark) were exposed to SNG, and fluorescent readings were taken usingthe EnSpire™ multimode plate reader with ex/em at 485 nm/535 nm.

### 2.8. LPO Analysis by Flow Cytometry

The Image-iT LPO kit was used to measure the lipid ROS levels through oxidation of the C-11-BODIPY 581/591 sensor, according to the manufacturer’s instructions. Briefly, after the treatment with SNG, the cells were washed with PBS, trypsinized, and centrifuged. The pellets were then stained with C11-BODIPY 581/591 (2 μM) for 30 min at 37 °C. Oxidation of the polyunsaturated butadienyl portion of the dye resulted in a fluorescence emission peak shift from ~590 nm to ~510 nm, detected using flow cytometric analysis (BD FACS Aria III; Becton Dickinson, Heidelberg, Germany). The median fluorescence intensity (MFI) was then quantitated using FlowJo V.10.1 software.

### 2.9. Determination of Total GSH

GSH levels were determined as detailed in the instructions from the manufacturer’s protocol. Briefly, SNG-treated cells were lysed in 150 μL of lysis buffer (5% sulfosalicylic acid). The lysates were centrifuged, and 50 μL of supernatant was mixed with 150 μL of assay buffer (potassium phosphate buffer, pH 7.0, containing 5 mM EDTA, 1.5 mg/mL DTNB (5,5′-dithiobis-(2-nitrobenzoic acid), and 6 U/mL glutathione reductase). To this mixture, 50 μL of (0.16 mg/mL) NADPH (nicotinamide adenine dinucleotide phosphate) in a potassium phosphate buffer was added, and the absorbance was measured at 412 nm using an EnSpire™ multimode plate reader (PerkinElmer, Waltham, MA, USA).

### 2.10. Measurement of Cellular Labile Iron

The metal sensor Phen Green SK diacetate (PGSK) was used to detect intracellular labile iron. Briefly, the cells were seeded in six-well plates (0.5 × 10^6^ cells/well) one day before the experiment. After SNG treatment, the cells were washed with PBS, trypsinized, and centrifuged. The pellets were then stained with 500 μL of PGSK (5 μM) in PBS and were transferred to FACS tubes by incubating for 30 min at 37 °C in the dark. The fluorescence profile of the sample was monitored using a BD FACS Aria III flow cytometry (Becton Dickinson, Heidelberg, Germany). The median fluorescence intensity (MFI) of PGSK staining was then quantitated using FlowJo V.10.1 software.

### 2.11. Statistical Analysis

Statistical analysis was performed using Graph Pad Prism 8.0 software. Data are shown as mean ± standard deviation (*n* = 3). Significance was analyzed by one-way ANOVA using the Bonferroni post hoc test. The asterisk (*) represents *p*-value < 0.05, double asterisk (**) represents *p*-value < 0.01, and the triple asterisk (***) represents *p*-value < 0.001. Differences were considered significant only when *p* < 0.05.

## 3. Results

### 3.1. SNG-Induces Apoptosis in HeLa Cells

**Figure 1:**
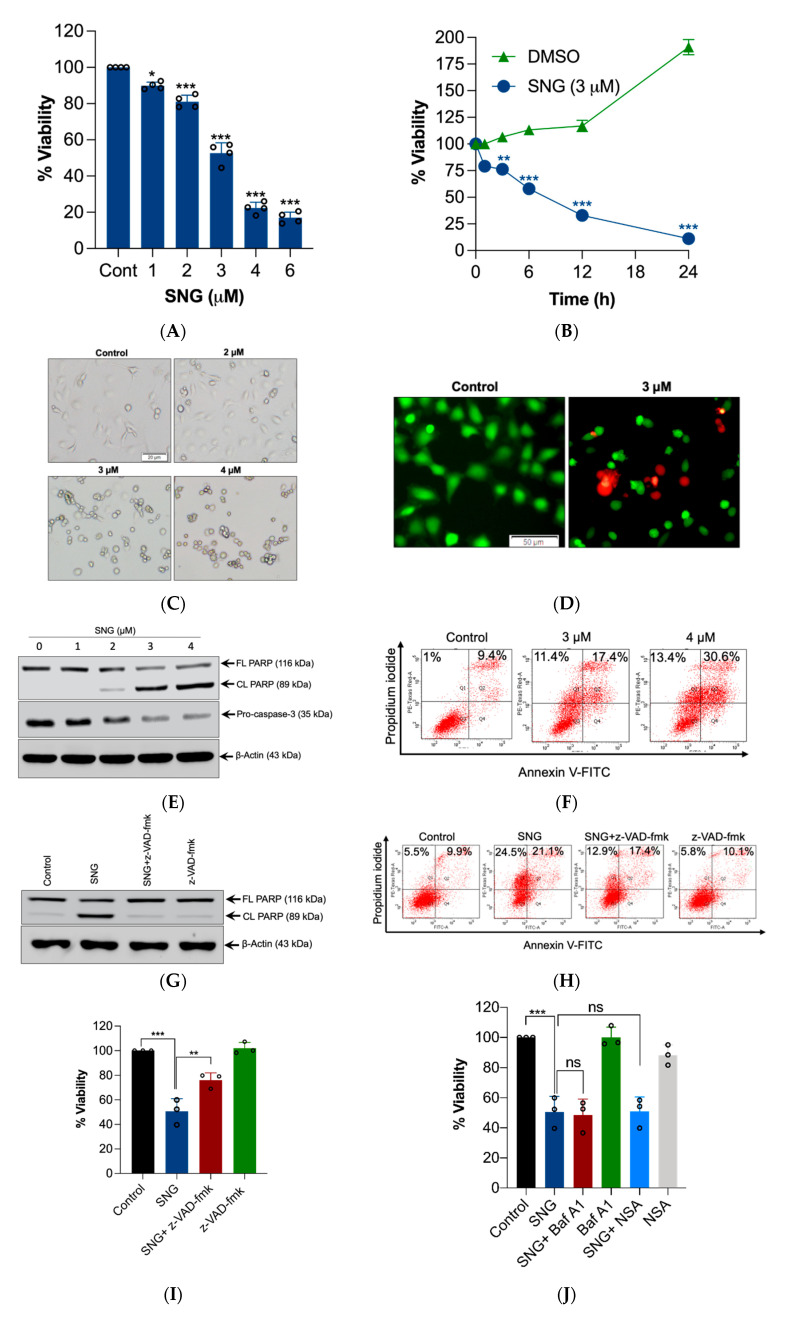
SNG-induced cell death is associated with features of apoptosis. HeLa cells were treated with (**A**) indicated concentrations of SNG for 6 h and (**B**) 3 μM SNG for the indicated time period. Cell viability was measured using an MTT assay. Data shown are means ± SD (*n* = 3) (* *p* < 0.05, ** *p* < 0.01, and *** *p* < 0.001) vs. the respective control. Cells were treated with indicated concentrations of SNG for 6 h. Following treatment, (**C**) morphological changes were assessed by microscopy, (**D**) live-dead assay was performed, and a (**E**) Western blot analysis of indicated proteins was performed. Actin was used as the loading control, and (**F**) apoptosis analysis by flow cytometry was done using Annexin V-FITC/PI staining. HeLa cells pretreated with z-VAD-fmk were treated with SNG (3 μM) for 6 h. Following treatment, (**G**) Western blot analysis of indicated proteins was performed. Actin was used as a loading control, (**H**) apoptosis analysis by flow cytometry using the Annexin V-FITC/PI staining, and (**I**) cell viability was assessed by the MTT assay. Data shown are mean ± SD (*n* = 3). ** *p* < 0.01 and *** *p* < 0.001. (**J**) HeLa cells were pretreated with Baf A1 or NSA were treated with SNG (3 μM) and cell viability was measured by the MTT assay. Data shown are mean ± SD (*n* = 3). *** *p* < 0.001. ns, no significance.

Initially, we tested the cytotoxicity of SNG on HeLa cells. SNG caused both a dose- and time-depended reduction in cell viability, as assessed by MTT assay (Figure 1A,B). Morphologically, the SNG-treated cell demonstrated cytotoxic features such as shrinkage, rounding, and partial detachment (Figure 1C). As changes in the cellular metabolism may affect the MTT assay, cell viability was also measured using the live/dead assay. SNG treatment caused significant ethidium homodimer staining (Red), while untreated cells were only stained by calcein (Green) (Figure 1D), suggesting that the live/dead assay data correlate to the MTT data.

Most of the cytotoxic agents kill cancer cells by inducing apoptosis. Therefore, we investigated whether SNG induces apoptosis in HeLa cells. HeLa cells were treated with various concentrations of SNG and a Western blot analysis of apoptosis markers was performed. A reduction in procaspase-3 and increased cleavage of its substrate PARP (critical indicators of caspase activation), particularly at high concentrations of SNG treatment, was observed (Figure 1E). Next, FACS analysis following Annexin V-FITC/PI staining was performed, which detects the externalization of phosphatidylserine on cells, a widespread method for the assessment of apoptosis. As shown in Figure 1F, apoptosis was significantly increased dose-dependently in HeLa cells treated with SNG relative to the control. Moreover, pan caspase inhibitor z-VAD-fmk completely negated SNG-induced PARP cleavage (Figure 1G) and Annexin V-FITC/PI staining (Figure 1H), yet only partially protected from the cell death (Figure 1I). This suggests the involvement of other modes of cell death in addition to apoptosis in HeLa cells.

Previously, we reported that SNG induces the simultaneous activation of apoptosis and autophagy in human malignant glioma cells [2]. Thus, we checked for the involvement of autophagy in SNG-treated HeLa cells. Pretreatment with Baf A1 (which blocks autolysosome acidification and autophagic proteolysis) [16] had no effect on SNG-induced cell death (Figure 1J). In addition, SNG-induced cell death was not inhibited by NSA, a commonly used necroptosis inhibitor, refuting the involvement of necroptosis in SNG-induced cytotoxicity (Figure 1J).

### 3.2. SNG-Induced Cell Death Was Associated with the Features of Ferroptosis

**Figure 2:**
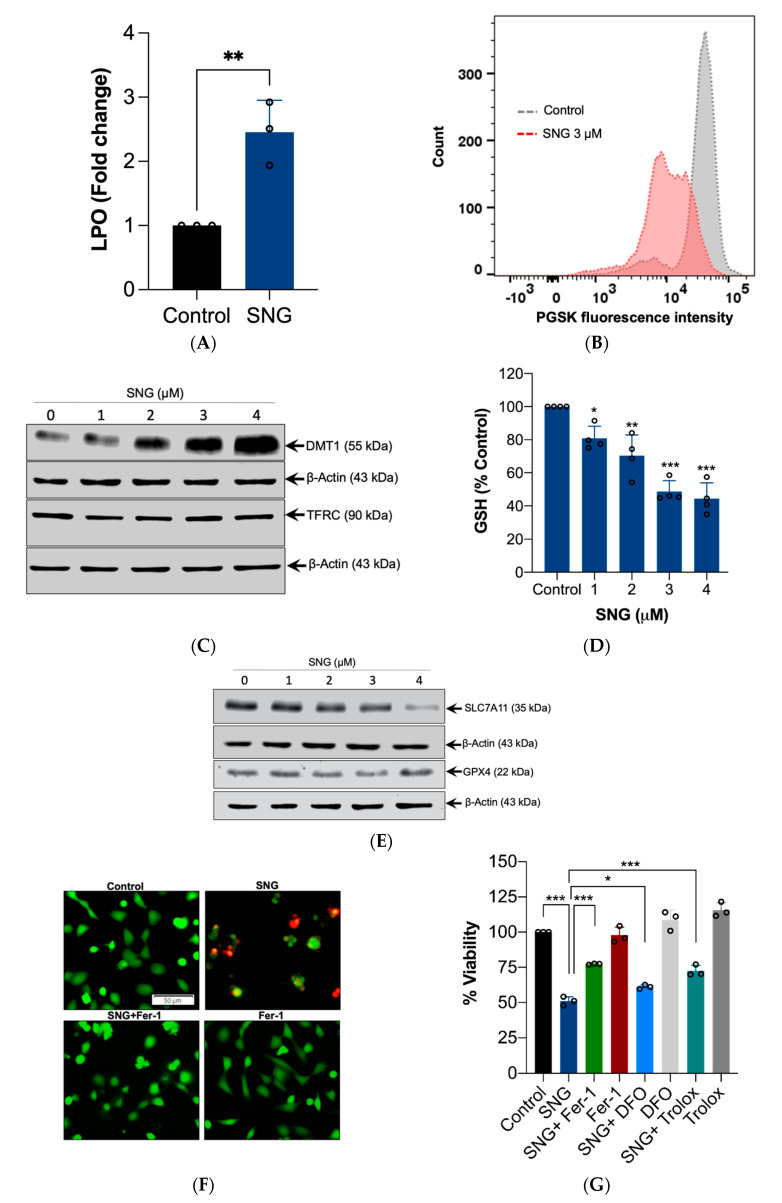
SNG induces the features of ferroptosis in HeLa cells. HeLa cells were treated with SNG. Following treatment, (**A**) LPO was determined by flow cytometry using C11-BODIPY 581/591 prob. Data shown are mean ± SD (*n* = 3). Significant differences, ** *p* < 0.01, (**B**) intracellular labile iron was determined by FACS using the fluorescent indicator PGSK, (**C**,**E**) and Western blot analysis of the indicated proteins was performed. Actin was used as a loading control, and the (**D**) GSH levels were measured. Data shown are means ± SD (*n* = 4) (* *p* < 0.05, ** *p* < 0.01, and *** *p* < 0.001 vs. control). HeLa cells pretreated with Fer-1, DFO, or Trolox were treated with SNG (3 μM). Following treatment, (**F**) a live/dead assay was performed and (**G**) cell viability was assessed using an MTT assay. Data shown are means ± SD (*n* = 3) (* *p* < 0.05 and *** *p* < 0.001).

As z-VAD-fmk only partially negated the SNG-induced cell death, we examined whether ferroptosis is involved in SNG-induced cytotoxicity. LPO, a key event of ferroptosis [11], was increased by SNG treatment (Figure 2A). Intracellular labile free iron is indispensable for LPO and ferroptosis execution [17]. Thus, we assessed intracellular labile free iron using the fluorescent indicator Phen Green SK, the fluorescence of which is quenched by iron. As expected, SNG treatment triggered a decrease in the proportion of Phen Green SK-positive cells (Figure 2B). It has been shown that Fe^3+^ is imported into the cells through the transferrin receptor (TFRC) and is then sequestered in the endosomes. In the endosome, Fe^3+^ is reduced to ferrous iron (Fe^2+^). The divalent metal transporter 1 (DMT1) mediates the release of Fe^2+^ from the endosome into a labile iron pool in the cytoplasm [18]. Therefore, iron accumulation in cells is closely related to TFRC and DMT1. To explore the functions of SNG in iron metabolic pathways, Western blot analysis for TFRC and DMT1 was carried out. SNG increased the expression of DMT1, but the expression of TFRC was not affected in HeLa cells (Figure 2C).

GSH is a physiologically defensive molecule that prevents LPO. GSH depletion is considered as the onset cause of ferroptosis [19]. SNG treatment significantly reduced GSH levels in HeLa cells (Figure 2D). SLC7A11 is a crucial constituent of the cystine/glutamate antiporter (system Xc^−^), which typically mediates the inflow of extracellular L-cystine, which is important for GSH synthesis [19]. Glutathione peroxidase 4 (GPX4) is a selenoenzyme responsible for diminishing phospholipid hydroperoxide through the oxidation of GSH [19]. It has been indicated that disturbed system Xc^−^ or GPX4 leads to ferroptosis. Therefore, the expressions of the SLC7A11 and GPX4 levels were tested. As shown in Figure 2E, SNG significantly reduced SLC7A11 levels with a negligible impact on the expression of GPX4. Collectively, these data suggest that SNG induces the features of ferroptosis through SLC7A11 downregulation, GSH reduction, labile iron pool accumulation, and LPO, which are the characteristic features of ferroptosis. Moreover, pretreatment with Fer-1 and Trolox (LPO inhibitors), and DFO (an iron-chelating agent), partially, but significantly, inhibited SNG-induced cytotoxicity in HeLa cells, suggesting that SNG, apart from inducing apoptosis, can also induce ferroptosis in HeLa cells (Figure 2F,G).

### 3.3. ROS Generation, Specifically H_2_O_2_ Triggers SNG-Induced Apoptosis and Ferroptosis in Human Cervical Cancer Cells

**Figure 3:**
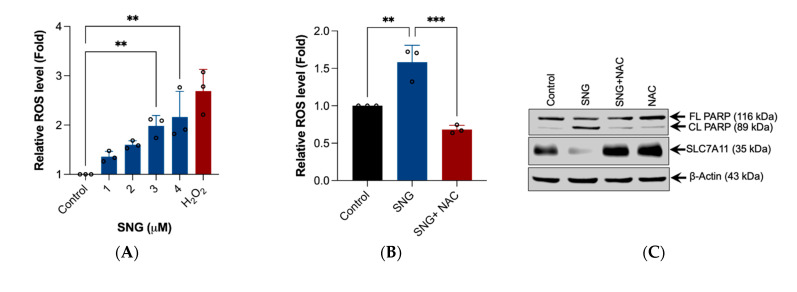

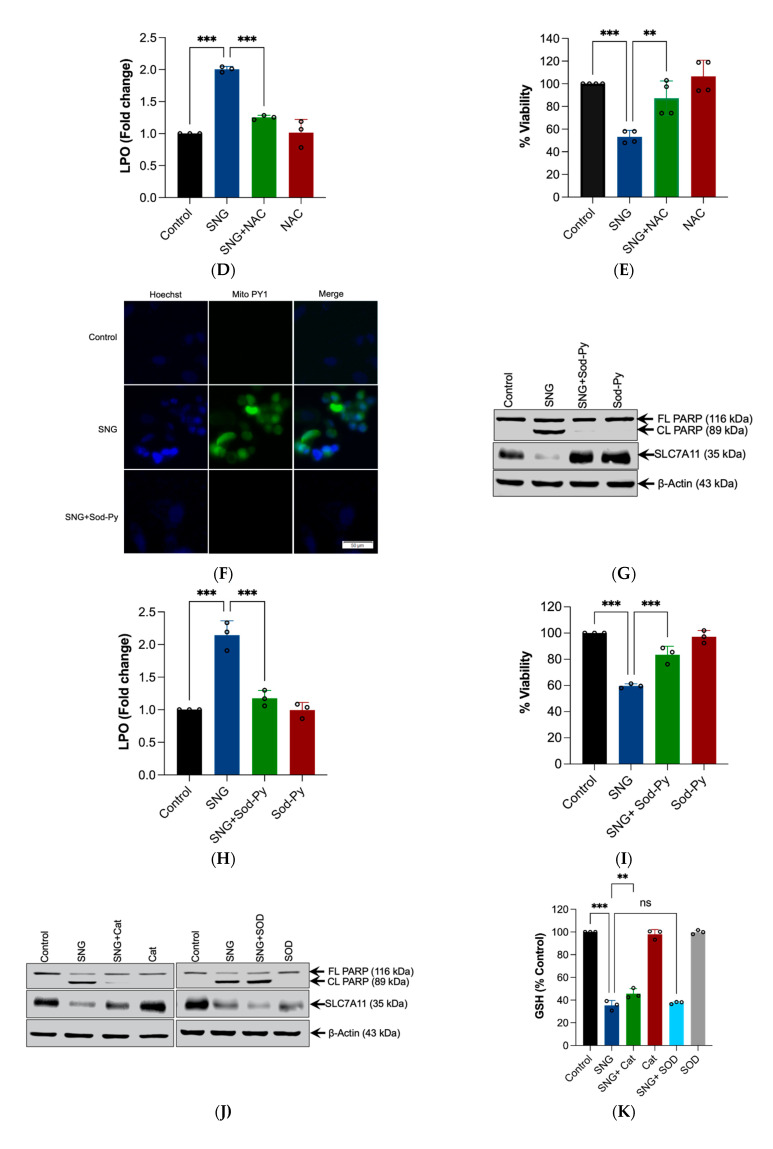

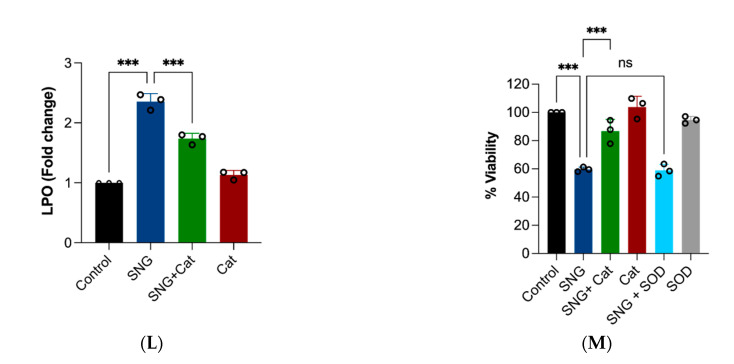
H_2_O_2_ is the ROS molecule responsible for SNG-induced apoptosis and ferroptosis in HeLa cells. Cells were treated with the indicated concentration of SNG for 1 h. Following treatment, (**A**) the cells were stained with DCFH-DA and analyzed using fluorometry. H_2_O_2_ (1 mM) was used as a positive control. Data shown are mean ± SD (*n* = 3), (** *p* < 0.01). The cells were pretreated with NAC for 1 h, followed by SNG treatment. Following treatment, (**B**) cells were stained with DCFH-DA and were analyzed by fluorometry. Data shown are mean ± SD (*n* = 3), ** *p* < 0.01 and *** *p* < 0.001, (**C**) Western blot analysis of indicated proteins were performed. Actin was used as a loading control, (**D**) LPO was determined by flow cytometry using the C11-BODIPY 581/591 prob. Data shown are mean ± SD (*n* = 3). Significant differences, *** *p* < 0.001, and (**E**) cell viability were assessed using an MTT assay. Data shown are means ± SD (*n* = 4) (** *p* < 0.01, and *** *p* < 0.001). (**F**) Cells were treated with MitoPY1 were stained with Hoechst 33,342 (1.5 µL; 10 mg/mL stock solution). After treatment with SNG in the presence or absence of Sod-Py, the fluorescence of MitoPY1 (green) and Hoechst (blue) was detected using fluorescent microscopy. Cells pretreated with Sod-Py were treated with SNG. After treatment, (**G**) the Western blot analysis of the indicated proteins were performed. Actin was used as a loading control, (**H**) LPO was determined by flow cytometry using the C11-BODIPY 581/591 prob. Data shown are mean ± SD (*n* = 3). Significant differences, *** *p* < 0.001, and (**I**) cell viability were assessed using an MTT assay. Data shown are means ± SD (*n* = 3) (*** *p* < 0.001). The cells pretreated with Cat or SOD were treated with SNG. Following the treatment, (**J**) Western blot analysis of the indicated proteins were performed. Actin was used as a loading control, and the (**K**) GSH levels were measured. Data shown are means ± SD (*n* = 3) (** *p* < 0.01 and *** *p* < 0.001), (**L**) LPO was determined by flow cytometry using the C11-BODIPY 581/591 prob. Data shown are mean ± SD (*n* = 3) (*** *p* < 0.001), and (**M**) cell viability was assessed using an MTT assay. Data shown are means ± SD (*n* = 3) (*** *p* < 0.001).

Studies have shown that oxidative stress is an important causative factor to induce apoptosis and ferroptosis [12]. Previously, we have shown that SNG-induces ROS-dependent apoptosis in leukemic and prostate cancer cells [3,9], and ROS-dependent autophagy in malignant glioma cells [2]. Therefore, we examined whether SNG induces ROS generation in HeLa cells. Treatment of HeLa cells with SNG resulted in a rapid and sustained DCFH-DA-derived fluorescence generation, suggesting the ROS generation (Figure 3A). In order to establish whether ROS has any role in SNG-induced apoptosis and ferroptosis, the cells were pretreated with ROS scavenger NAC, followed by SNG treatment. The cells were then examined for apoptosis and ferroptosis. Pretreatment with NAC completely abrogated the SNG-induced ROS generation (Figure 3B), PARP cleavage and SLC7A11 downregulation (Figure 3C), LPO (Figure 3D), and loss of viability (Figure 3E), demonstrating that SNG induces ROS-dependent apoptosis and ferroptosis.

The mitochondrial respiratory chain is a major source of ROS production within most mammalian cells [12]. Therefore, to directly assess whether SNG induces mitochondria-derived H_2_O_2_, a mitochondrial-specific fluorescent probe (MitoPY1) that selectively turned on fluorescence enhancement in response to H_2_O_2_ was used. As shown in Figure 3F, an increase in the MitoPY1-derived fluorescence was observed in the cells treated with SNG. To confirm that the increase in MitoPY1-derived fluorescence was attributable to H_2_O_2_, Sod-Py (a potent intracellular scavenger of H_2_O_2,_) was used [20]. Pretreatment with Sod-Py completely blocked the SNG-induced MitoPY1-derived fluorescence (Figure 3F), features of apoptosis and ferroptosis (Figure 3G,H), and loss of viability (Figure 3I). Furthermore, pretreatment with catalase (Cat) (a specific scavenger of H_2_O_2_) significantly blocked SNG-induced apoptosis and ferroptosis (Figure 3J–L) and loss of viability (Figure 3M), while SOD (a specific scavenger of superoxide; O_2_^−^) failed to do so (Figure 3J,K,M). Altogether, these results clearly demonstrate that H_2_O_2_ generation plays an essential role in SNG-induced apoptosis and ferroptosis in human cervical cancer cells.

### 3.4. Crosstalk between Apoptosis and Ferroptosis in SNG-Induced Cell Death

**Figure 4:**
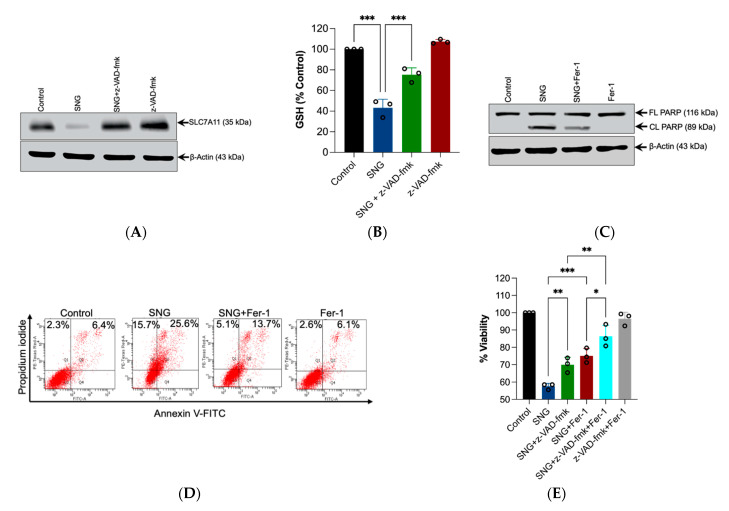
Crosstalk between apoptosis and ferroptosis in SNG-induced cell death. Cells were treated with SNG (3 μM) in the presence or absence of z-VAD-fmk for 6 h. Following treatment, (**A**) Western blot analysis of the indicated proteins were performed. Actin was used as a loading control and the (**B**) GSH levels were measured. Data shown are means ± SD (*n* = 3) (*** *p* < 0.001). Cells were treated with SNG (3 μM) in the presence or absence of Fer-1 for 6 h. Following treatment, (**C**) Western blot analysis of the indicated proteins was performed and actin was used as the loading control, (**D**) apoptosis was analyzed by Annexin V-FITC/PI staining. (**E**) The cells were treated with SNG (3 μM) in the presence or absence of a combination of z-VAD-fmk and Fer-1 for 6 h. Following treatment, cell viability was assessed using an MTT assay. Data shown are means ± SD (*n* = 3) (* *p* < 0.05, ** *p* < 0.01, and *** *p* < 0.001).

Apoptosis can be interrelated to ferroptosis [21]. As SNG induces both apoptosis and ferroptosis in human cervical cancer cells, we examined their interrelation. Figure 4A,B shows that apoptosis inhibitor z-VAD-fmk significantly inhibited the features of ferroptosis, such as SLC7A11 down regulation and GSH depletion. Similarly, th ferroptosis inhibitor Fer-1 significantly prevented SNG-induced apoptosis (Figure 4C,D). These results revealed that both apoptosis and ferroptosis induction are positively correlated with each other. Next, the cell viability assay was analyzed in the cells co-treated with SNG and z-VAD-fmk combined with Fer-1. As expected, the combination of z-VAD-fmk with Fer-1 demonstrated considerable protection from SNG-induced cytotoxicity (Figure 4E), indicating that apoptosis and ferroptosis regulate each other in a positive feedback manner during SNG-induced cell death in human cervical cancer cells.

## 4. Discussion

In recent years, the therapeutic potential of medicinal plants as a source of novel and promising anticancer agents has gained widespread attention among cancer biologists. Herbal constituents not only exert a diverse biological activity, but also have a reduced overall toxicity compared with chemically synthesized drugs [22]. Uncovering the function and mechanisms of these natural molecules has become one of the research hotspots [23]. In the present study, we report that SNG, a naturally occurring benzophenanthridine alkaloid exhibited anti-tumor activities via synergistically inducing both apoptosis and ferroptosis in HeLa cells. We have made further progress towards understanding the sub-cellular mechanism through which SNG triggers cancer cell death, by identifying that ROS, in particular H_2_O_2_, is responsible for SNG-induced cytotoxicity.

Inducing apoptosis is one of the main methods to inhibit the growth of tumor cells [24]. We and others have previously shown that SNG induces apoptosis in a number of different cancers [1,5,9]. In this study, we report that SNG induces apoptosis in HeLa cells, as evidenced by caspase activation, PARP cleavage, and annexin V-FITC/PI staining. However, caspase inhibitor z-VAD-fmk only partially prevented SNG-induced cell death, suggesting that other caspase-independent pathways in SNG-induced human cervical cancer cytotoxicity might exist. Consequently, we observed that SNG also induces features of ferroptosis in HeLa cells. SNG caused increased labile iron pool and LPO, while it reduced the expression of SLC7A11 and GSH levels. Moreover, ferroptosis inhibitors (Fer-1, Trolox, and DFO) significantly negated SNG-induced cell death, confirming that the ferroptosis pathway might also be involved in determining the cytotoxic activity of SNG in HeLa cells. In line with our finding, recently, SNG was found to inhibit the growth and metastasis of non-small cell lung cancer by inducing ferroptosis [25].

Extensive evidence in the literature validates that ROS can effectively induce cell death, such as apoptosis and ferroptosis [12]. Several lines of evidence in our study demonstrate that ROS plays a vital role in SNG-induced death in human cervical cancer cells. First, SNG induces rapid ROS generation. Second, pretreatment of cells with ROS scavengers almost completely abolishes SNG-induced PARP cleavage, SLC7A11 down-regulation, and LPO. Third, the cytotoxic effect of SNG is significantly abolished in the presence of ROS scavengers. We also identified specific ROS responsible for the SNG-induced cytotoxicity. Pretreatment with specific H_2_O_2_ scavengers (Sod-Py and Cat) provided significant protection from loss of viability, PARP cleavage, and SLC7A11 down-regulation, but the O_2_—scavenger (SOD) failed to do so.

**Figure 5:**
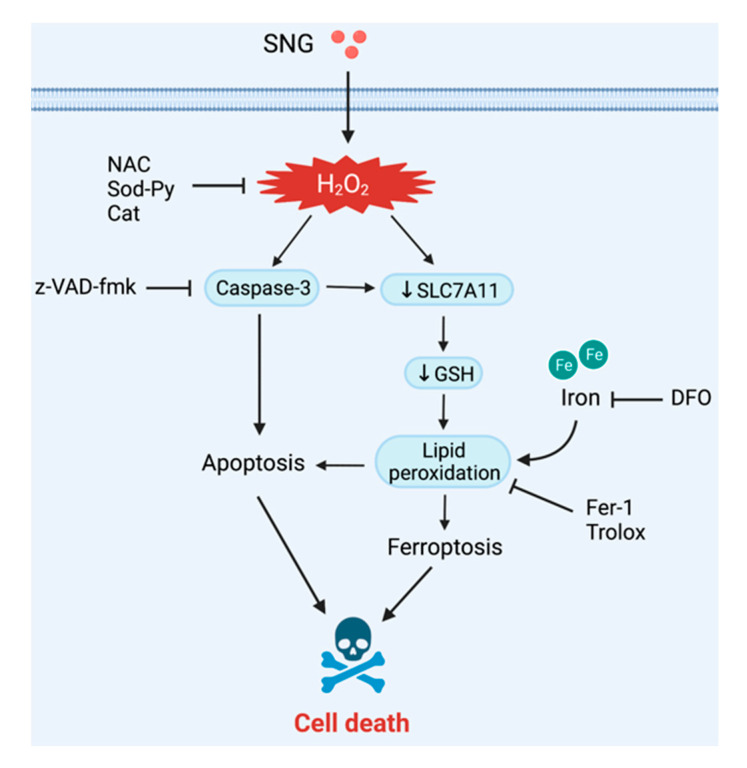
Schematic representation of SNG-mediated cell death. SNG induced the accumulation of intracellular ROS, promoting caspase activation and apoptosis. In addition, ROS further triggered down-regulation of SLC7A11 and GSH depletion and LPO, resulting in the activation of ferroptosis.

To date, emerging evidence indicates that ferroptosis often shares common pathways with other types of cell death. In light of this, recent studies have revealed that the ferroptotic agent-induced ER stress response plays an important role in the cross-talk between ferroptosis and apoptosis [21]. From the above experimental results, we propose that SNG induces ROS-mediated cervical cancer cell death through the crosstalk between two mechanisms of programmed cell death pathways—apoptosis and ferroptosis (Figure 5). This crosstalk mechanism has been further supported by three important results. First, ROS inhibitors completely reversed ferroptotic and apoptotic cell death. Second, inhibiting ferroptosis abolished the features of apoptosis and cell death. Third, suppressing apoptosis with z-VAD-fmk not only inhibited the features of SNG-induced apoptosis, but also inhibited the features of SNG-induced ferroptosis. In conclusion, our study provided potential mechanistic pathways for SNG-induced human cervical cancer cell death. These compelling results expanded our understanding of the clinical applications of SNG-based therapy against cervical cancer.

## Data Availability

The data of the present study are available from the corresponding author upon reasonable request.

## Abbreviations

BafA1	bafilomycin A1
Cat	catalase
DCFH-DA	2′,7′-dichlorodihydrofluorescein diacetate
DFO	deferoxamine
DMEM	dulbecco’s modified essential medium
DMT1	divalent metal transporter 1
DMSO	dimethyl sulfoxide
FBS	fetal bovine serum
Fer-1	Ferrostatin-1
GPX4	glutathione peroxidase 4
GSH	glutathione
H_2_O_2_	hydrogen peroxide
LPO	lipid peroxidation
MTT	3-[4,5-dimethylthiazol-2-yl]-2,5-diphenyl tetrazolium bromide
NAC	N-acetyl cysteine
NSA	necrosulfonamide
PGSK	phen Green SK diacetate
ROS	reactive oxygen species
SLC7A11	solute carrier family 7 member 11
SNG	sanguinarine
SOD	superoxide dismutase
Sod-Py	sodium pyruvate
TFRC	transferrin receptor
